# The incremental burden of pain in patients with depression: results of a Japanese survey

**DOI:** 10.1186/s12888-015-0488-8

**Published:** 2015-05-07

**Authors:** Jeffrey Vietri, Tempei Otsubo, William Montgomery, Toshinaga Tsuji, Eiji Harada

**Affiliations:** 1 Kantar Health, Health Outcomes Practice, Via Paleocapa 7, 20121 Milan, Italy; 2 Tokyo Shinjuku Medical Center, Tokyo, Japan; 3 Eli Lilly Australia Pty Ltd, West Ryde, Australia; 4 Shionogi & Co., LTD., Osaka, Japan; 5 Eli Lilly Japan K.K., Kobe, Japan

**Keywords:** Depression, Pain, Painful physical symptoms, Quality of life, Work productivity, Health care use

## Abstract

**Background:**

Major depressive disorder (MDD) is a chronic mental illness which affects an estimated 3% of the Japanese population. Many patients with MDD report painful physical symptoms, and research outside of Japan suggests such patients may represent a subtype of depression which is more severe and difficult to treat. There is no evidence available about the characteristics or incremental burden of these patients in Japan. The objective of this study was to quantify the incremental burden of physical pain among individuals in Japan diagnosed with depression.

**Methods:**

Data for individuals age 18 and older who reported a physician diagnosis of depression were obtained from the Japan National Health and Wellness Survey (NHWS). Respondents who also reported physical pain were matched to respondents who did not report pain using propensity scores and compared using bivariate statistics. Measures included Patient Health Questionnaire (PHQ-9) for depression severity, Medical Outcomes Study 12-Item Short Form Survey Instrument (SF-12v2) for health-related quality of life, the Work Productivity and Activity Impairment (WPAI) for work and activity impairment, and 6-month report of health care use.

**Results:**

Individuals with depression who reported physical pain had higher PHQ-9 depression scores (14.3 vs. 11.1, p<0.001), lower health-related quality of life (Mental Component Summary score [MCS] 29.1 vs. 32.0, p<0.01; Physical Component Summary score [PCS] 43.0 vs. 47.2, p<0.001; health utility [SF-6D] 0.567 vs. 0.613, p<0.001), more presenteeism (46.3% vs. 36.8%, p<0.01), more overall work impairment (51.4% vs. 42.3%, p<0.01), more activity impairment (55.4% vs. 43.9%, p<0.001), and reported using more health care provider visits in the prior 6 months (17.7 vs. 12.8, p<0.01) as well as hospitalizations (1.7 vs. 0.8, p<0.05) relative to propensity-score matched controls without pain. Absenteeism (13.1% vs. 11.4%, p=0.51) and emergency room visits (0.31 vs. 0.35, p=0.76) were not significantly different between the two matched groups.

**Conclusions:**

Individuals whose depression is accompanied by physical pain have a higher burden of illness than those whose depression does not include physical pain. Clinicians should take the presence of pain into account and consider treating both the physical and emotional symptoms of these patients.

## Background

Major Depressive Disorder (MDD) is a severe recurring illness associated with depressed mood, loss of interest or pleasure, feelings of guilt or low self-worth, disturbed sleep or appetite, psychomotor agitation or slowing, low energy, poor concentration, and risk of suicide. Depression has a considerable impact on both patients with the condition as well as on broader society [1-3]. MDD is one of the most common mood disorders, with a 12-month prevalence of approximately 3% in Japan according to the World Health Organization Composite International Diagnostic Interview [4]. While the prevalence of mood disorders such as MDD is considerably lower in Japan than in many Western countries, there are significant cohort differences in susceptibility to mood disorders, with higher prevalence in younger generations [5]. The higher lifetime prevalence expected for these younger people suggests an increase in the societal burden of depression is likely in the future. Patients suffering from depression sometimes present with painful physical symptoms as well [6], which many exacerbate this burden.

As is the case with depression, chronic pain conditions have been found to lead to substantial disability [7-10]. Furthermore, previous research has shown that a high proportion of patients with depression experience a range of painful physical symptoms [11,12]. The subset of depressed patients who experience such pain symptoms appears to have a particularly high level of disability. Relative to depressed patients without comorbid pain, individuals with depression and pain have greater limitations in daily activities [11,12], have worse health related quality of life (HRQoL) [11-15], and are more likely to be unemployed due to disability [11,16]. Patients with both depression and pain also use more inpatient and outpatient health care resources and are more expensive to treat [11,14,17-20]. In fact, it has been suggested that patients with depression and painful physical symptoms may constitute a distinct subpopulation of patients which is more difficult to treat [21].

The connection between painful physical symptoms and greater depression severity, lower quality of life, and greater health care use was recently demonstrated in a multi-country prospective study of individuals presenting with an acute depressive episode in East Asia [22]. In that study, painful physical symptoms were reported by approximately half of the patients enrolled, and such symptoms were associated with clinically important decrements in quality of life at 3 months post-baseline [22]. This decrement was maintained after adjusting for covariates including baseline level of depression [23]. Similarly, worse pain symptoms were associated with lower remission rates of depression [24]. However, Japan was not included in the aforementioned prospective study, and little real-world data from Japan has been reported regarding either the incremental burden of physical pain on patients with depression or the burden on the Japanese health care system. Research describing patient-reported health outcomes such as HRQoL and impairment to work and daily activities among these individuals is particularly scarce.

Therefore the objective of this study was to assess the incremental burden of physical pain in real-world patients with depression in Japan, including the association between the presence of physical pain and the severity of depression symptoms.

## Methods

### Data source

Data were provided by the Japan National Health and Wellness Survey (NHWS; Kantar Health, NY, USA), an annual internet-based survey of the general population aged 18 and older. Potential respondents for the NHWS were selected from an opt-in survey panel through random sampling stratified by age and gender to match the Japanese population aged 18 years and older. This project included two years of Japan NHWS data, with 25,000 respondents collected in 2010 and 30,000 in 2011. These two years of data were collected independently of one another but combined to provide a more robust sample size for the current analysis. Respondents who participated in both years of the survey were identified and only the more recent response was included in the analyses. In addition to membership in the survey panel, respondents were required to read and write Japanese, be at least 18 years old, and provide informed consent. All information was collected through self-report. The protocol and questionnaire for the NHWS were reviewed and approved by Essex Institutional Review Board (Lebanon, New Jersey, USA).

### Measures

#### Depression

The NHWS includes questions on experience, diagnosis, and treatment of a broad variety of medical conditions, one of which is depression. Those who indicated they had experienced depression in the prior 12 months were then asked if their depression had been diagnosed by a doctor. Respondents who indicated a diagnosis by a doctor were considered to have depression and were asked additional details regarding the year of diagnosis, the type of physician, and whether they were currently using a prescription medication for their depression. The diagnostic criteria used and setting of the diagnosis were not assessed in the survey.

#### Depression symptoms

Depression symptoms were assessed using the PHQ-9 [25], a validated scale used to screen for depression and to assess its severity, translated into Japanese. This scale measures depression through the frequency of anhedonia, depressed mood, sleep disturbance, lack of energy, appetite disturbance, negative self-feelings, difficulty concentrating, psychomotor retardation or agitation, and thoughts of self-harm in the prior two weeks. The severity of depression was assigned according to the standard cutoff scores: 5, 10, 15, and 20 points for mild, moderate, moderately severe, and severe depression, respectively. The total score can also be considered as a continuous variable. A single-item measure of the interference of these symptoms is also included in the instrument. The PHQ-9 was not included in the 2011 Japan NHWS, and comparisons of depression severity therefore included fewer respondents than other measures which were common across both 2010 and 2011. In addition to the PHQ-9, respondents also rated the severity of their depression as mild, moderate, or severe.

#### Painful physical symptoms

The NHWS asks respondents to indicate whether they have experienced pain in the 12 months preceding the survey (yes or no); those who indicated experiencing pain were considered to have painful physical symptoms. In order to avoid attributing pain from obvious physical causes to painful symptoms associated with depression, respondents who reported pain caused by broken bones, cancer, dental problems, menstrual cycle, post herpetic neuralgia, surgery or a medical procedure, or phantom limb pain (neuropathic pain following amputation) were excluded from the analyses.

#### Health-related quality of life (HRQoL)

The Japanese language version of the revised Medical Outcomes Study 12-Item Short Form Survey Instrument (SF-12v2) was used to measure HRQoL. This is a multipurpose, generic HRQoL instrument comprising of 12 questions [26] developed from the widely used SF-36v2 [27]. Two summary scores calculated from this measure were used: the physical component summary (PCS) score, an index of overall physical functioning, and the mental component summary (MCS) score, which is an index of mental and emotional health. Scores can be interpreted relative to the US population average of 50 with a standard deviation of 10, with higher scores indicating better HRQoL.

Responses to the SF-12v2 were also used to generate health state utilities according to the SF-6D algorithm, a preference-based, single index measure for health using general population values [28]. The SF-6D index has interval scoring properties and yields summary scores on a theoretical 0–1 scale (with an empirical floor of 0.3). Higher scores indicate better (more preferred) health status, with 1 being equivalent to perfect health.

#### Work productivity and activity impairment

Work productivity was assessed using the Japanese language version of the Work Productivity and Activity Impairment (WPAI) questionnaire, which assesses absenteeism (work time missed), presenteeism (impairment while at work), overall work productivity impairment (a combination of absenteeism and presenteeism), and activity impairment (impairment in daily activities) due to health problems over the prior seven days [29]. All are reported as percentages, with higher numbers indicating greater impairment. Only respondents who reported being full-time or part-time employed provided data for absenteeism, presenteeism, and overall work impairment. All respondents provided data for activity impairment.

#### Health care resource use

Health care resource use is assessed in the NHWS as all-cause health care provider (HCP) visits, emergency room (ER) visits, and hospitalizations in the 6 months prior to the survey. HCP visits were calculated by summing the self-reported number of visits to specific types of health care providers (e.g., general internist, psychiatrist, allergist, dentist, nurse, etc.).

### Analysis

The analysis was conducted in three phases. First, patients with depression were compared according to the presence of physical pain using t-tests for continuous variables and chi-square tests for categorical variables. This analysis served to identify patient characteristics that were associated with physical pain and describe the differences in outcomes, irrespective of the presence of potentially confounding patient characteristics across the two groups.

The second step of the analysis was the assignment of propensity scores. Propensity scores were calculated using a binary logistic regression predicting the presence of pain within the sample reporting a diagnosis of depression. The regression incorporated age, sex, completion of university education, household income category, body mass index (BMI) category, cigarette smoking, alcohol use, exercise, length of depression diagnosis, type of diagnosing physician, and Charlson comorbidity index (CCI) as predictors. These variables were selected for inclusion either because of their potential relationships with outcomes, observed differences in unmatched comparisons, or both. Variables that differed according to the presence of pain that could be considered outcomes, including employment status, depression severity, HRQoL, work and activity impairment, and health care resource use were not used as predictors in the regression. The predicted values from this regression were used as propensity scores to match each respondent with depression and pain to a single respondent whose depression did not include pain using a greedy matching algorithm.

The third and final step in the analysis was comparison of the two matched samples (depression with and without pain) using independent-samples t-tests and chi-square tests. These comparisons provide an incremental burden of physical pain in depression after eliminating measured confounders.

## Results

### Sample characteristics

**Figure 1:**
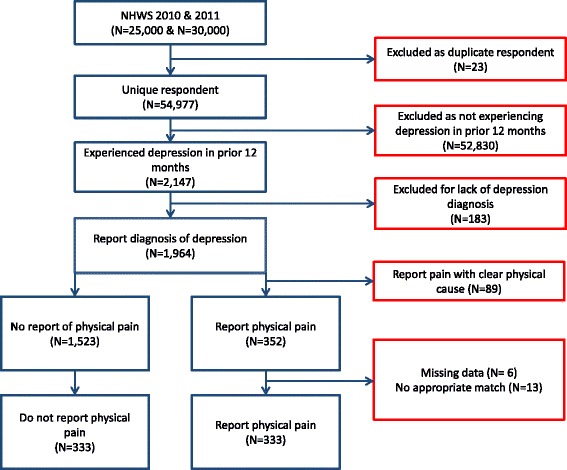
Disposition of NHWS respondents in matched comparisons.

The disposition of Japan NHWS respondents is presented in Figure 1. A total of 54,977 individuals completed the Japan NHWS across 2010 and 2011, and 2,147 of those respondents indicated experiencing depression in the prior 12 months. Of these, 1,964 indicated a doctor diagnosis of depression. Eighty-nine (89) were excluded due to the source or type of pain experienced, resulting in 1,875 respondents with a physician diagnosis of depression included in the analysis. After exclusions for source of pain, physical pain was reported by 18.8% (n=352) of the sample. The average respondent was 40.6 years old and had been diagnosed with depression 6.2 years prior to the survey. A total of 80% reported current treatment for depression with a prescription medication. The sample was 52.6% males, 63.2% were employed, 50.6% had completed a 4-year college degree or greater, and 44.3% reported being married or living with a partner.

### Unmatched comparisons

Demographic and health characteristics are presented in Table 1. Pain was associated with different demographic characteristics among those reporting a diagnosis of depression. Those reporting pain were more likely to be women, less likely to have a 4-year university degree, less likely to be employed, and tended to fall into the lower income categories than those without pain. The proportion of respondents using alcohol, smoking cigarettes, and exercising in the prior month were similar across the two groups, though respondents who reported pain differed in the distribution of BMI categories and had higher CCI scores.

**Table 1:** **Sociodemographic and health characteristics by the presence of physical pain**

	Depressed without pain (N=1523)		Depressed with pain (N=352)		
	n	%	n	%	p-value
Female	687	45.1%	202	57.4%	<0.001
Marital status					0.039
Married or living with partner	671	44.1%	160	45.5%	
Single, never married	702	46.1%	143	40.6%	
Divorced	150	9.8%	49	13.9%	
Annual household income					0.070
Less than ¥ 3,000,000	382	25.1	114	32.4	
¥ 3,000,000 to ¥ 4,999,999	371	24.4	71	20.2	
¥ 5,000,000 to ¥ 7,999,999	342	22.5	71	20.2	
¥ 8,000,000 or more	295	19.4	67	19.0	
Decline to answer	133	8.7	29	8.2	
University degree or greater	799	52.5%	150	42.6%	0.001
Employed	981	64.4%	204	58.0%	0.024
BMI categories					0.010
Underweight	176	11.6%	56	15.9%	
Normal	870	57.1%	190	54.0%	
Overweight	286	18.8%	78	22.2%	
Obese	123	8.1%	14	4.0%	
Decline to answer	68	4.5%	14	4.0%	
Drinks alcohol	1065	69.9%	255	72.4%	0.351
Smokes cigarettes	475	31.2%	117	33.2%	0.456
Vigorous exercise in past month	624	41.0%	153	43.5%	0.392
	Mean	SD	Mean	SD	p-value
Age	40.3	12.2	41.5	12.1	0.118
BMI (n=1460, 339)	23.32	5.07	22.73	4.86	0.052
CCI	0.22	0.69	0.53	2.12	0.006*

Note: p-values for comparison of frequencies are from omnibus Pearson chi-square test; p-values for comparison of means are from t-test; asterisk indicates p-value adjusted for violation of homogeneity of variance assumption; BMI: Body mass index; CCI: Charlson comorbidity index.

Depression characteristics are presented in Table 2. Regardless of whether pain was present or not, approximately four out of every five respondents in the sample had been diagnosed by a psychiatrist, and a similar proportion was currently using a prescription for depression. Those with pain had been diagnosed with depression an average of one year longer, and their depression was more severe. This finding of greater depression severity among those with pain was consistent whether assessed through self-rated severity, PHQ-9 score, or the distribution of PHQ-9 severity categories. Patients with pain also rated their depression as making it harder to work or function than those without pain.

**Table 2:** **Depression characteristics by the presence of physical pain**

	Depressed without pain (N=1523)		Depressed with pain (N=352)		
	n	%	n	%	p-value
Diagnosing physician (n=1,481; 338)					0.027
General internist	156	10.5%	53	15.7%	
Psychiatrist	1250	84.4%	268	79.3%	
Other	75	5.1%	17	5.0%	
Currently use prescription	1229	80.7%	274	77.8%	0.226
Depression severity (self-rated)					0.005
Mild	614	40.3%	117	33.2%	
Moderate	697	45.8%	165	46.9%	
Severe	212	13.9%	70	19.9%	
Depression severity categories (PHQ-9; n=824; 185)					<0.001
None	162	19.7%	13	7.0%	
Mild	239	29.0%	47	25.4%	
Moderate	149	18.1%	40	21.6%	
Moderately severe	140	17.0%	41	22.2%	
Severe	134	16.3%	44	23.8%	
Problems made it hard to work/function (PHQ-9; n=769; 182)					0.012
Not difficult at all	126	16.4%	16	8.8%	
Somewhat difficult	426	55.4%	101	55.5%	
Very difficult	139	18.1%	35	19.2%	
Extremely difficult	78	10.1%	30	16.5%	
	Mean	SD	Mean	SD	p-value
Length of depression diagnosis (years) (n=1481; 338)	6.0	5.7	7.1	7.1	0.010*
PHQ-9 total score (n=824; 185)	11.3	7.4	14.1	7.1	<0.001

Note: p-values for comparison of frequencies are from omnibus Pearson chi-square test; p-values for comparison of means are from t-test; asterisk indicates p-value adjusted for violation of homogeneity of variance assumption.

Patients with depression and pain also had worse outcomes in terms of quality of life and functioning as measured by the SF-12v2 and WPAI, and reported more frequent use of health care resources (Table 3). Those with pain had decrements of 2.8 points on the MCS score and 4.5 points on the PCS score relative to those who did not report pain, and SF-6D health utility scores were .05 points lower than those who had depression without pain. Impairment to work and daily activities were significantly greater among those with pain relative to those without pain, and HCP visits were also more frequent, though ER visits and hospitalizations were not significantly different.

**Table 3:** **General patient-reported outcomes by the presence of physical pain**

	Depressed without pain (N=1523)		Depressed with pain (N=352)		
	Mean	SD	Mean	SD	p-value
HRQoL					
Mental Component Summary	31.9	11.9	29.1	11.2	<0.001
Physical Component Summary	47.5	8.2	43.0	8.8	<0.001
Health utility score (SF-6D)	0.615	0.099	0.567	0.093	<0.001
Work impairment					
Absenteeism (%) (n=914; 191)	14.8	29.1	13.7	26.4	0.649
Presenteeism (%) (n=895; 191)	38.0	26.7	46.4	25.7	<0.001
Overall work impairment (%) (n=914; 191)	45.5	31.6	51.7	29.2	0.009*
Activity impairment (%)	45.1	27.7	55.3	25.8	<0.001*
Health care use (past 6 months)					
HCP visits	12.7	12.2	17.6	17.2	<0.001*
ER visits	0.29	1.63	0.32	1.42	0.745
Hospitalizations	1.16	6.95	1.74	7.37	0.178*

Note: HRQoL: Health-related quality of life; ER: Emergency room; HCP: Health care provider; p-values for comparison of frequencies are from omnibus Pearson chi-square test; p-values for comparison of means are from t-test; asterisk indicates p-value adjusted for violation of homogeneity of variance assumption.

### Matching procedure

A total of 56 respondents (14 with pain and 42 without) were excluded from the logistic regression because of missing data for length of diagnosis. Higher odds of having pain were associated with female sex, higher CCI score, longer duration of depression, and lack of a university degree (Table 4). The presence of pain also varied with BMI category. Five respondents with pain were not able to be matched to a control without pain, resulting in a total of 333 respondents with both depression and pain and 333 matched controls.

**Table 4:** **Odds ratios of reported pain among individuals with depression in Japan**

		95% confidence limits		
	Odds ratio	Lower	Upper	p-value
Age (1 year)	0.999	0.987	1.011	0.856
Female	1.601	1.219	2.104	<0.001
Marital status (overall)				0.326
Married/living with partner	Reference			
Single, never married	0.830	0.607	1.134	0.134
Divorced	1.122	0.748	1.682	0.295
Household income (overall)				0.157
Less than ¥ 3,000,000	Reference			
¥ 3,000,000 to ¥ 4,999,999	0.673	0.471	0.963	0.333
¥ 5,000,000 to ¥ 7,999,999	0.679	0.466	0.988	0.384
¥ 8,000,000 or more	0.677	0.457	1.001	0.395
Decline to answer	0.821	0.511	1.319	0.670
University degree or greater	0.744	0.572	0.967	0.027
BMI category (overall)				0.010
Underweight	Reference			
Normal	0.772	0.533	1.119	0.520
Overweight	1.048	0.672	1.634	0.011
Obese	0.372	0.191	0.724	0.008
Decline to answer	0.624	0.313	1.246	0.604
Drinks	1.200	0.908	1.587	0.200
Smokes	1.095	0.836	1.435	0.509
Exercises	1.141	0.889	1.466	0.300
CCI	1.398	1.197	1.634	<0.001
Diagnosing doctor (overall)				0.241
General internist	Reference			
Psychiatrist	0.735	0.514	1.052	0.288
Other	0.784	0.417	1.474	0.757
Length of diagnosis (1 year)	1.024	1.004	1.045	0.018

Note: p-value is from Wald chi-square test in binary logistic regression; BMI: Body mass index; CCI: Charlson comorbidity index.

### Matched comparisons

There were no significant differences between the two matched groups in sociodemographics and health characteristics (all p > 0.40; Table 5) or in the depression-specific variables included in the regression (i.e., type of diagnosing doctor and length of diagnosis, both p>0.74; Table 6). Though not included in the matching equation, current use of a prescription medication for depression was similar across those with and without pain (Table 6).

**Table 5:** **Sociodemographic and general health characteristics in the matched sample by the presence of physical pain**

	Depressed without pain		Depressed with pain		
	(N=333)		(N=333)		
	n	%	n	%	p-value
Female	184	55.3%	191	57.4%	0.584
Marital status					0.818
Married	159	47.7%	151	45.3%	
Single, never married	129	38.7%	136	40.8%	
Divorced	45	13.5%	46	13.8%	
Annual household income					0.986
Less than ¥ 3,000,000	110	33.0%	109	32.7%	
¥ 3,000,000 to ¥ 4,999,999	62	18.6%	67	20.1%	
¥ 5,000,000 to ¥ 7,999,999	67	20.1%	66	19.8%	
¥ 8,000,000 or more	66	19.8%	62	18.6%	
Decline to answer	28	8.4%	29	8.7%	
University degree or greater	150	45.0%	140	42.0%	0.434
Employed	198	59.5%	194	58.3%	0.753
BMI categories					0.808
Underweight	56	16.8%	51	15.3%	
Normal	181	54.4%	181	54.4%	
Overweight	76	22.8%	74	22.2%	
Obese	12	3.6%	14	4.2%	
Decline to answer	8	2.4%	13	3.9%	
Drinks alcohol	250	75.1%	241	72.4%	0.428
Smokes cigarettes	117	35.1%	112	33.6%	0.683
Vigorous exercise in past month	144	43.2%	145	43.5%	0.938
	Mean	SD	Mean	SD	p-value
Age	41.7	12.6	41.5	11.9	0.815
BMI (n=318; 320)	22.7	4.0	22.8	4.9	0.701
CCI	0.37	1.11	0.42	0.95	0.524

Note: p-values for comparison of frequencies are from omnibus Pearson chi-square test; p-values for comparison of means are from t-test; BMI: Body mass index; CCI: Charlson comorbidity index.

**Table 6:** **Depression characteristics by presence of physical pain (matched comparisons)**

	Depressed without pain		Depressed with pain		
	(N=333)		(N=333)		
	n	%	n	%	p-value
Diagnosing physician					0.921
General internist	52	15.6%	50	15.0%	
Psychiatrist	266	79.9%	266	79.9%	
Other	15	4.5%	17	5.1%	
Currently use prescription	260	78.1%	265	79.6%	0.635
Depression severity (self-rated)					0.184
Mild	128	38.4%	110	33.0%	
Moderate	156	46.8%	159	47.7%	
Severe	49	14.7%	64	19.2%	
Depression severity (PHQ-9; n=194; 177)					<0.001
None	42	21.6%	11	6.2%	
Mild	48	24.7%	45	25.4%	
Moderate	44	22.7%	38	21.5%	
Moderately severe	33	17.0%	41	23.2%	
Severe	27	13.9%	42	23.7%	
Problems made it hard to work/function (PHQ-9; n=180; 175)					0.088
Not difficult at all	28	15.6%	16	9.1%	
Somewhat difficult	102	56.7%	96	54.9%	
Very difficult	33	18.3%	34	19.4%	
Extremely difficult	17	9.4%	29	16.6%	
	Mean	SD	Mean	SD	p-value
Length of depression diagnosis (years)	7.0	6.7	6.8	6.5	0.742
PHQ-9 total score (n=198; 175)	11.1	7.3	14.3	7.1	<0.001

Note: p-values for comparison of frequencies are from omnibus Pearson chi-square test; p-values for comparison of means are from t-test.

However, differences in severity of depression between those with and without pain remained significant in the matched comparisons when measured by the PHQ-9 total score or PHQ-9 severity categories. In contrast, self-ratings of severity did not differ (Table 6). The ratings of how much the depression made it hard to work or function (which is not asked of respondents without depression symptoms on the PHQ-9) showed a non-significant trend for more interference among those with pain (p=.09; Table 6).

The pattern of poorer HRQoL, more work and activity impairment, and greater health care use among those with painful physical symptoms noted in the unmatched comparison was confirmed in the matched comparisons (Table 7). HRQoL was worse in those with depression and pain compared to those with depression alone, with the mean MCS score 2.9 points lower, mean PCS score 4.2 points lower, and mean health utility score .046 points lower in those with pain. Presenteeism was higher by 10% among those with pain, and impairment in non-work activities was 12% greater in absolute terms. As in the unmatched comparisons, those with pain visited HCPs more frequently, with 4.9 more visits on average in a 6-month period, and 0.9 more hospitalizations during the same time, though rates of ER use were similar in those with and without physical pain.

**Table 7:** **General patient-reported outcomes by the presence of physical pain (matched comparisons)**

	Depressed without pain		Depressed with pain		
	(N=333)		(N=333)		
HRQoL	Mean	SD	Mean	SD	p-value
Mental Component Summary	32.0	11.9	29.1	11.3	0.001
Physical Component Summary	47.2	8.4	43.0	8.8	<0.001
Health utility score (SF-6D)	0.613	0.097	0.567	0.094	<0.001
Work impairment					
Absenteeism (%) (n=188; 181)	11.4	24.9	13.1	25.7	0.510
Presenteeism (%) (n=181; 191)	36.8	26.6	46.3	25.5	<0.001
Overall work impairment (%) (n=191;182)	42.3	30.4	51.4	29.0	0.003
Activity impairment (%)	43.9	27.1	55.4	25.7	<0.001
Health care use (past 6 months)					
HCP visits	12.8	12.7	17.7	17.2	<0.001
ER visits	0.35	1.87	0.31	1.36	0.758
Hospitalizations	0.78	4.11	1.72	7.27	0.041

Note: HRQoL: Health-related quality of life; ER: Emergency room; HCP: Health care provider; p-values are from t-test.

## Discussion

The current study demonstrates that individuals in Japan with depression and painful physical symptoms have both more-severe depression and impaired health outcomes relative to those whose depression does not include painful symptoms. A broad variety of health outcomes are impacted, including HRQoL, work productivity impairment, and health care resource use. The impact of physical pain among these patients remained significant after propensity score matching equated the groups on a variety of potential confounders, including age, sex, education, level of income, length of diagnosis, type of diagnosing physician, and comorbidity burden, among others.

Consistent with previous research [22,24], the current study found an association between physical pain and the severity of depression, which was reflected in both the MCS score, which is a broad index of mental and emotional health, and the PHQ-9 score, which is specific to depression. These findings further support the idea that depression which includes painful physical symptoms is a more severe subtype of depression [21]. The difference in MCS scores was just below the 3-point threshold often considered the minimally important difference (MID) for this measure [27]. Not surprisingly, physical pain showed an impact outside of the measures designed to assess mental and emotional well-being. The difference in PCS scores observed was greater than the 3-point MID threshold. The .046 difference observed in SF-6D utility scores is also substantial, and greater than the .030 interval often considered the MID for that measure [30]. The impact of pain was not limited to HRQoL, as those with pain reported 1.26 times the presenteeism according to the WPAI, 1.22 times the overall work impairment, and 1.26 times the activity impairment reported by those without pain. Health care use was likewise elevated, with nearly 5 more HCP visits on average per patient during the 6-month recall period and one additional hospitalization among those with pain relative to those without pain.

The prevalence of pain among those with depression was considerably lower in the current study (18%) than was reported among other East Asians (52%) [22,24]. It is not clear to what extent the difference reflects differences in the populations studied versus differences in measurements used in the studies. The aforementioned prospective study found differences in the prevalence of painful physical symptoms across the countries included in the study using a common definition, which was a threshold of greater than or equal to 2 points on the Somatic Symptoms Inventory pain scale [31]. However, that scale is not used in the NHWS. Instead, the current study required the patient to identify pain as condition they had experienced in the prior 12 months, and this difference in assessment may have been partially responsible for the lower prevalence reported here. Differences in measures used also prevent comparing the magnitude of the differences between those with and without pain between this study and the previous one, as the measures used to assess depression severity and HRQoL also differed.

Like any study, the current analysis has important limitations. Details regarding the diagnosis of depression were not available in the survey data. Indeed, some individuals in the present study may not have met diagnostic criteria for MDD at the time of the survey. Other NHWS respondents may have been suffering from depression but were not included here due to lack of diagnosis being required for inclusion in this study. While it was not possible to corroborate the diagnosis self-reported in NHWS with medical records, the high prevalence of self-reported prescription use for depression and the results of the PHQ-9 provide additional evidence that the sample studied here is suffering from depression. Approximately 80% of the sample was currently using a prescription medication for depression, and the vast majority of patients in this study had symptoms of depression, with 82.7% of respondents rated as having at least mild depression symptoms on the PHQ-9. Likewise, the definition of pain in the current study differed from previous studies, and no specific pain scale was administered to all respondents of the NHWS. Incorporating a scale such as the Somatic Symptoms Inventory to screen individuals for pain may have identified additional respondents with depression who were experiencing pain but who did not indicate pain as one of their conditions in the survey. The cross-sectional and correlational nature of the survey did not allow for assessment of the time course of the physical symptoms, and it was not clear whether individuals had painful physical symptoms at the onset of the emotional components of the depressive episode or whether the pain developed at another time. The propensity scores were generated by a regression analysis, the results of which may have differed if different variables were included, or if the variables used in the regression were coded in a different way. Finally, while the matching procedure equalized the groups on a number of measured potential confounders, it is possible that an unmeasured variable differed across the matched groups and was responsible for some portion of the differences observed.

## Conclusion

In conclusion, the presence of physical pain among depression patients in Japan appears to have an effect similar to that seen in other countries, where it is associated with more-severe depression, treatment resistance, worse health outcomes, greater use of health care resources, and lower productivity. Clinicians caring for these patients should take the presence of physical pain into account and consider treating both the physical and emotional symptoms of depression.
